# Contraception and abortion knowledge, attitudes and practices among adolescents from low and middle-income countries: a systematic review

**DOI:** 10.1186/s12913-018-3722-5

**Published:** 2018-11-29

**Authors:** Margarate Nzala Munakampe, Joseph Mumba Zulu, Charles Michelo

**Affiliations:** 1 Department of Health Policy and Management, School of Public Health, University of Zambia, Nationalist Road, P.O Box 50110, Lusaka, Zambia; 2 Department of Health Promotion and Education, School of Public Health, University of Zambia, Lusaka, Zambia; 3 Department of Epidemiology & Biostatistics, School of Public Health, University of Zambia, Lusaka, Zambia; 4 Strategic Centre for Health Systems Metrics & Evaluations (SCHEME), Department of Epidemiology & Biostatistics, School of Public Health, University of Zambia, Lusaka, Zambia

**Keywords:** Adolescents, Contraception, Abortion, Systematic review

## Abstract

**Background:**

Adolescents face significant barriers to contraception access and utilization that result in adverse health effects of early pregnancy and childbirth. Unsafe abortions continue to occur partly due to failure to prevent pregnancies, with Sub-Saharan Africa contributing the most significant burden of all unsafe abortions among young people globally, of which a quarter occurs in those aged 15–19 years. We aimed to conduct a systematic review of the contraceptive and abortion knowledge, attitudes and practices of adolescents in low and middle-income countries to increase the understanding of the sexual and reproductive health dynamics that they face.

**Methods:**

Literature searches from 6 databases; PubMed, Science Direct, Google Scholar, BioMed Central, CINAHL, MEDLINE, were conducted, covering the period from 1970 to 2016 and concerning the adolescents aged 15–19 years and 21 studies were read and analyzed using thematic analysis.

**Results:**

Limited knowledge about sexual and reproductive health among adolescents was a significant cause of reduced access to contraception and safe abortion services, especially among unmarried adolescents. Reduced access to reproductive health services for some resulted in extreme methods of contraception and abortion such as the use of battery acid and crushed bottles. Despite all adolescents having limited access to information and services, girls faced more consequences such as being blamed for pregnancy or dealing with the effects of unsafe abortions. Parents, health workers, and teachers were cited as trusted sources of information but often received the most information from peers and other family members instead, and the girls mostly confided in their aunties, cousins and peers while the boys resorted to peers, media and even pornography.

**Conclusion:**

The reported observations suggest severe limitations in the access to safe and effective methods of contraception and safe abortion services. There is a need for an urgent response in reducing the “unmet needs” for contraception and to improve access to contraception, abortion information, and services in this group. Interventions which target the involvement of parents and teachers should be considered, to carry one wholesome message to the adolescents.

## Background

Article 14 of the Maputo Protocol on the Rights of Women in Africa reaffirms women’s rights to sexual and reproductive health; which include control of their fertility, choice of family planning methods, and access to safe abortion services on relatively liberal indications [1]. Nevertheless, contraception and safe abortion continue to be highly contested issues in most countries around the world. Unsafe abortion, evidence of a high unmet need for family planning and contraception continues to take place at high rates, not the least among adolescents with 57% of all unsafe abortions in sub-Saharan Africa found in the age-group 15–24, which is higher than in other regions. The most vulnerable segment – the girls from 15 to 19 years - account for some 25% of all unsafe abortions in Africa [2]. This vulnerability points to the urgency of addressing the unmet need for contraception in this group.

Adolescents face challenges with contraception and access to safe abortion. One-sixth of the women in the reproductive age group are adolescents aged 15 to 19 and about half of the pregnancies that occur among adolescents in this age group in developing regions are unintended [3]. In about 52 countries, it was revealed that sexually active never-married women have high levels of unmet need for contraception, with the highest level being among adolescents aged 15 to 19 [4]. Youths, who are at risk of unsafe abortion, do not even have full access [5] to reproductive health information and services because health providers usually shun them and are not treated well because they are not expected to be engaging in sexual activity at their age. Approximately 90% of abortion-related and 20% of pregnancy-related morbidity and mortality, along with 32% of maternal deaths, could be prevented by the use of effective contraception [6].

Studies have revealed various barriers to contraceptive use among adolescents in low and middle-income countries, LMICs and such obstacles include lack of, or limited knowledge, lack of sexuality education and limited access to services; high risk of misperceptions; and harmful social norms surrounding premarital sexual activity and pregnancy [7, 8]. It has been documented that sexual activity within or outside marriage for adolescents can lead to adverse outcomes, amplified by their limited access to services [9]. The strong societal influence on how adolescents should live their lives and how adolescents live their lives causes a lot of confusion for the adolescents, especially among peers [10].

While several efforts are striving to understand adolescents’ knowledge, attitudes and practices regarding contraception and safe abortion, systematic reviews on this matter and related contexts, remain limited. Understanding this field is essential to provide sufficient response to their sexual and reproductive health needs. We set out to conduct a systematic review on knowledge, attitudes and practices around contraception and abortion to identify any gaps that may generally be critically important to increase the understanding of the sexual and reproductive health dynamics that many adolescents around the world face.

## Methods

The main aim of this systematic review was to explore contraception and abortion knowledge, attitudes and practices among adolescents from LMICs. `.

### Search strategy

Using a strategy from previous studies [11], we initially searched using three search engines (Science Direct, Google Scholar, and PubMed) using generic terms. After abstracts from each of the three search engines were retrieved and read, the search terms were revised to add more relevant words and similar terminologies, and three more search engines were added (BioMed Central, CINAHL, and MEDLINE). The specific search terms included were: experiences OR attitudes OR perceptions, qualitative OR quantitative OR phenomenology OR phenomenological OR interviews OR discussions OR ethnography AND contraceptive OR contraception OR family planning OR pregnancy OR pregnant AND adolescent OR adolescence OR teen OR teenage OR teenager OR young woman OR young women OR young people OR young female OR young male OR young men OR girl OR girls OR boys AND Abortion OR induced abortion OR termination of pregnancy.

### Inclusion and exclusion criteria

Peer-reviewed articles from 1970 to 2016 were reviewed on contraception and abortion knowledge, attitudes and practices of adolescents. The review included perceptions, and perspectives on contraception, contraceptives and abortion or either. This period was selected to capture perceptions over time. All studies were qualitative or had a qualitative component in them which enabled the review of the adolescents’ perspectives on contraception and abortion in their different socio-cultural environments. Qualitative methods included ethnographic methods or phenomenological; focus group discussions and interviews, semi-structured questionnaires. Quantitative methods had to have looked into factors influence decision-making, perceptions of contraception and determinants of abortion decisions but should have included a qualitative component for triangulation. The reference lists of selected articles were reviewed, and the full texts of potentially interesting studies were examined. Included articles had to have reported on adolescents aged 15 to 19 years old, or the age group 15 to 19 needed to be part of the sample included in the study. Articles based on either male-only or female-only studies or a combination of both male and female adolescents were included. Only articles based on research conducted in LMICs were included, and all articles were published in English.

### Study selection and quality assessment

**Fig. 1:**
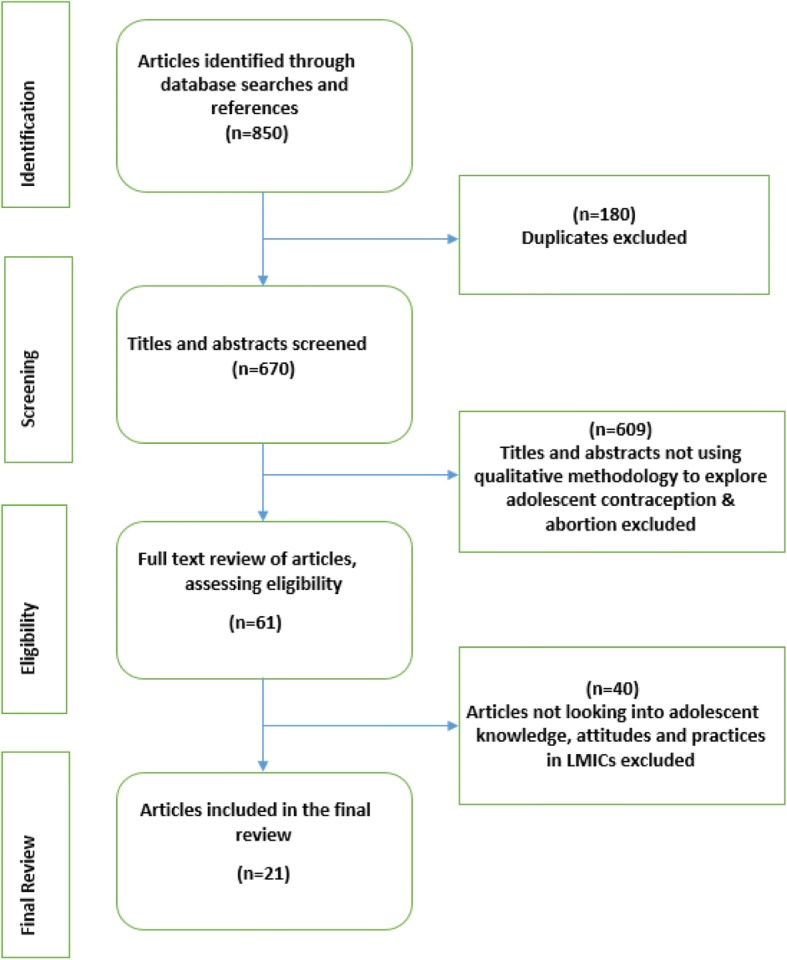
PRISMA Flow Chart

Out a vast amount of data on adolescents and contraception and abortion, 21 documents were selected and reviewed and analyzed, using the PRISMA guidelines suggested by Moher et al., 2009 [11, 12]. This selection was based on the study description, location, methods, and sample size. The database search yielded **850** articles. After removal of duplicates, **670** articles remained with 180 duplicates removed. Title review followed, and **128** articles remained with 542 articles with titles that did not contain sought-after information even if they had the key search terms in them. The following step was an abstract review where the methodologies were also reviewed. A total of 67 articles were removed. After full-text review or 61 articles, 25 were retained, and 36 were excluded because, at this point, we sought articles that focused adolescents. Finally, of the 25 articles, four were removed because they were based on studies from high-income countries. The final review was on 21 papers that reported contraception and abortion knowledge, attitudes and practices among adolescents in LMICs. These studies covered the period 2001 to 2015. Fig. 1 shows the summary of how the papers were selected.

Data were analyzed using thematic analysis [11], in NVivo 10 software. This analysis allowed for the exploration of the data; identifying different themes around the knowledge, attitudes and practices of the adolescents on issues of contraception and abortion. A code list was developed comprising of broad themes and smaller analytical themes. The development of themes continued throughout the coding process to allow for other emerging themes to be captured. There was no predetermined code structure, and so all the themes were emergent from the literature reviewed. We also documented the findings by gender (male and female adolescents), environment (rural and urban adolescents), and education status (in and out of school adolescents) and marital status (married and unmarried adolescents) to bring out the differences among different categories of adolescents.

### Findings

#### Review description

Overall, 21 studies from the period 2001 to 2015 were included in the final review. These were arranged by author and year of publication, the title of the article, country, methods and the sample characteristics (see Table 1). The results are presented based on the major themes that emerged from the analysis and synthesis. These include knowledge, attitudes and practices around contraception and abortion in LMICs. Findings of the studies are also presented according to gender, environmental and marital status and education level differences.

**Table 1:** Summary of study characteristics: contraception and abortion knowledge, attitudes and practices among adolescents from low and middle-income countries

Author & Date		Title of Article	Country	Methods	Sample	Key Findings
1	Wood & Jewkes, 2006 [26]	Blood Blockages and Scolding Nurses: Barriers to Adolescent Contraceptive Use in South Africa	South Africa: Limpopo District (rural setting)	Qualitative methods 35 IDIs 5 FGDs (3–5) Purposive sampling	Gender: Female Sample size: 35 adolescents and 14 nurses Age 14–20	Adolescent sexuality needs to be acknowledged to address the sexual needs of adolescents
2	Webb, 2000 [27]	Attitudes to ‘Kaponya Mafumo’: the terminators of pregnancy in urban Zambia	Zambia 5 urban districts Lusaka, Kitwe, Ndola, Livingstone, Chipata	Mixed Methods 1100 School Narratives 20 FGDs (10–15) Medical records Urban	Gender: Female & Male Sample size: 1500; pupils, nurses Age: 10–24	Staff attitudes, lack of sufficient information about services, and few targeted interventions for the different categories of adolescents led adolescents to access clandestine reproductive health services. There was a need to increase access to information and services among adolescents.
3	Sowmini, 2013 [28]	Delay in termination of pregnancy among unmarried adolescents and young women attending a tertiary hospital abortion clinic in Trivandrum, Kerala, India	India (Urban & rural settings)	Qualitative methods 34 IDIs	Gender Female Sample size: 34 Age: 10–24 Unmarried	This study revealed that many adolescents were in consensual sexual relationships with older males, some even experiencing pressure to engage in sexual activity. Knowledge and information and access to reproductive health services were meager, even among the sexually active adolescents.
4	Schuster, 2010 [29]	Women’s experiences of the abortion law in Cameroon: “What really matters.”	Cameroon (Urban setting)	Qualitative methods 4 IDIs Triangulated with hospital records and data from 65 other interviews.	Gender: Female Sample size:4 Age: 15–21	Many adolescents who were in relationships with older men were at risk of being pressurized into engaging in sexual activity and had little control over their fertility. There was also ambiguity about the law and what services were available for the adolescents. With such conditions, some ended up resorting to illegal abortion.
5	Nzioka, 2001 [30]	Perspectives of adolescent boys on the risk of unwanted pregnancy and sexually transmitted infections: Kenya	Kenya (Rural setting)	Qualitative methods 8 FGDs	Gender: Male Sample size: 90 Age: 15–19	Most adolescents had restricted access to contraception such as condoms because they felt this would disclose their sexual activity which they worked hard to conceal. They also needed more information about contraception and abortion including counselling and referral to facilities that were confidential and anonymous.
6	Ritcher & Mlambo, 2005 [31]	Perceptions of Rural teenagers on teenage pregnancy	South Africa	Qualitative methods 32 IDIs with adolescents	Gender: Male and female Sample size: 32 Age: 13–19	Most teens who fell pregnant did not intend to do so and as such; there were many misconceptions about pregnancy, sex and contraceptives. They attributed factors such as age, knowledge and skill to pregnancy. The risk of increased health problems due to lack of information needs to be addressed with more information and specialised services for the adolescents.
7	Macintyre et al., 2015 [32]	From disease to desire, pleasure to the pill: A qualitative study of adolescent learning about sexual health and sexuality in Chile	Chile	Qualitative methods 4 FGDs and 20 IDIs with adolescents and 7 IDIs with key informants	Gender: Male and Female Sample size: 51 Age: 16–19	Many advances in sexual and reproductive rights have been reported; the study suggested that many taboos surrounding access to services were broken leaving an enabling environment for knowledge dissemination. However, challenges discussing sexual violence and emergency contraception were still reported and needed to be addressed.
8	Kennedy et al., 2014 [33]	“These issues aren’t talked about at home”: a qualitative study of the sexual and reproductive health information preferences of adolescents in Vanuatu	South Pacific	Qualitative methods using 66 FGDs with adolescents and 12 IDIs Key informants	Gender: Male and female Sample size: 353 Age: 15–19	Comprehensive sexuality education to adolescents was seen to have lifelong protective effects on the adolescents’ health. The wide information gap among the adolescents could, therefore, be reduced with early sexuality education, through strengthening structures that offer this information.
9	Kennedy et al., 2013 [34]	“Be kind to young people, so they feel at home”: a qualitative study of adolescents’ and service providers perceptions of youth-friendly sexual and reproductive health services in Vanuatu	South Pacific	Qualitative methods using 66 FGDs with adolescents and 12 IDIs Key informants	Gender: Male and female Sample size: 353 Age: 15–19	Adolescents were reported to face many barriers to accessing sexual and reproductive health services such as lack of confidentiality, skilled providers, and cultural barriers. Findings showed that most of the adolescents needed to be educated more to reduce the knowledge gap and to increase adolescent friendly service provision.
10	Ganatra & Hirve, 2002 [35]	Induced abortions among adolescent women in rural Maharashtra, India	India	Mixed methods: Survey of 1717 FDGs and IDIs with 197 adolescents and other Key informants	Gender: Female Sample size: 1717/ 197 Age: < 20	Adolescents faced significant barriers to access to contraception and abortion; such as untrained providers and lack of confidentiality. Unmarried adolescents had a greater unmet need, leading them to access informal services.
11	Char et al., 2011 [36]	Assessing young unmarried men’s access to reproductive health information and services in rural India	India	Mixed methods 4 FGDs and survey of 216 men	Gender: Male Sample size: 354 Age:17–22	Rural adolescents were willing to receive information and services, but these were both lacking in their setting. Interventions need to focus on different categories of adolescents to improve access to information and services
12	Both & Samuel, 2014 [37]	Keeping silent about emergency contraceptives in Addis Ababa: a qualitative study among young people, service provider and key stakeholders	Ethiopia	Qualitative methods, using observations, IDIs with young people, IDIs with key informants and key stakeholders	Gender: Males and Females Sample size: 112 Age: 15 to 29	Health care providers were significantly associated with how adolescents access and the amount of reproductive health information available to them. Health care provider attitudes need to be looked into to increase access to services by the adolescents.
13	Barua & Kurz, 2001 [38]	Reproductive health-seeking by married adolescent girls in Maharashtra	India	Mixed methods including survey and IDIs	Gender: Male and Female Sample size: 466 Age: 15 to 19	Married adolescents’ contraception decisions were mostly influenced by families and significant others. Though abortions and contraception were more acceptable among these adolescents, they have reduced access due to their reduced decision making power.
14	Ilika A., & Igwegbe, A., 2004 [39]	Unintended pregnancy among unmarried adolescents and young women in Anambra state. Southeast Nigeria	Nigeria	Qualitative methods using IDIs.	Gender: Female Sample size: 136 Age: 15 to 19	Adolescents presented high-risk sexual behaviour, unwanted pregnancy, unsafe abortions, Sexually Transmitted Infections, and HIV/AIDS. Discrimination from community, families and health care providers reduced access to services that could avert these peculiar challenges. A need for increased access to information and services was therefore stressed.
15	Otoide V. O., et al., 2001 [40]	Why Nigerian adolescents seek abortion rather than contraception: evidence from focus group discussions	Nigeria	Qualitative study, FGDs	Gender: Female Sample size: 149 Age: 15 to 24	The effective educational strategy to improve sexual and reproductive health information levels among the adolescents was stressed, to correct the deep-rooted misconceptions about contraception and reproductive health among adolescents.
16	Silberscmidt, M & Racsh, V, 2001 [41]	Adolescent girls, illegal abortions and sugar daddies in Dar es Salaam: Vulnerable victims and active social agents	Tanzania	Qualitative, IDIs	Gender: Female Sample size: 51 Age: 15 to 19	Despite international recognition of the importance of addressing adolescent sexual and reproductive health, access to clandestine abortions remains a challenge, pointing out to a need to increase efforts to address these issues among adolescents.
17	Dahlback et al., 2007 [42]	Unsafe induced abortions among adolescent girls in Lusaka	Zambia	Qualitative methods; IDIs	Gender: Female Sample size: 34 Age: 13 to 19	Despite induced abortion being legal for over 30 years, unsafe abortions, especially among adolescents, remain high. Limited information, limited access to services, stigma attached to premarital pregnancy, were outlined as factors that will continue to lead adolescents into accessing clandestine abortions if not addressed adequately.
18	Nguyen et al., 2006 [43]	Knowledge of contraception and sexually transmitted diseases and contraception practices amongst young people in Ho Chi Minh City, Vietnam	Vietnam	Qualitative study; IDIs	Gender: Male and Female Sample size: 16 Age: 15 to 24	Lack of youth-friendly sexual and reproductive health information and services have been attributed to an increased risk of unwanted pregnancy and unsafe abortion. A need to address their ever-changing sexuality to meet their access and knowledge needs was seen.
19	Nobelius et al., 2010 [44]	Sexual and reproductive health information sources preferred by out-of-school adolescents in rural southwest Uganda	Uganda	Qualitative methods; FGDs and 10 IDIs	Gender: Male and Female Sample size: 31 Age: 13 to 19	Most interventions to increase knowledge of sexual and reproductive health were mostly targeted towards adolescents in school, leaving a much greater information gap among the out of school adolescents, who were more vulnerable due to reduced access to services. A need for interventions for out of school adolescents was seen.
20	Okereke, 2010 [45]	Assessing the prevalence and determinants of adolescents’ unintended pregnancy and induced abortion in Owerri, Nigeria	Nigeria	Mixed methods using a survey, FGDs and IDIs with key informants	Gender: Female Sample size:555 Age: 15 to 19	Religious doctrines place many adolescents in a position to denounce utilisation of sexual and reproductive health services due to perceptions of the community as well as personal moral dilemmas, exposing them to reproductive health problems such as unintended pregnancy and abortion.
21	Plummer et al., 2006 [46]	Abortion and suspending pregnancy in rural Tanzania: An ethnography of young people’s beliefs and practices	Tanzania	Qualitative: participants observations in 9 villages for 7 weeks; FGDs and Interviews	Gender: Female and Male Sample size: Age:15 to 27	Strict legal sanctions led to adolescents accessing clandestine abortion services to terminate a pregnancy. Low usage of family planning and contraception were seen as the underlining factor that contributed to these risks.

### Knowledge about contraception and abortion

Knowledge of contraception is a determinant of contraceptive usage. Adolescents were asked what they knew about contraception and abortion, and generally, the studies showed that adolescents had poor, limited, incomplete and sometimes wrong knowledge/information about contraception and abortion (1, 2, 3, 5, 4, 10, 8, 9, 11, 15, 16, 17, 18). They also had poor sexuality or sexual and reproductive health information, in particular, about how conception occurred and this caused misconceptions or incorrect information regarding conception. Also, they could not effectively adopt safer pregnancy prevention strategies and other good reproductive health practice due to the low knowledge (4, 18). In these studies, the adolescents reported that even though they were taught about sexuality in schools, they lacked detailed information about how to protect themselves from infections, for example, adolescents complained that they were informed about the importance of using condoms but not how to wear them when using them (7, 8, 9).

Furthermore, the adolescents in these studies mentioned that the manner in which sexuality information was packaged was not sufficient for their information needs; health care providers shunned them or parents talked about the risks of having sex rather than love and affection for one’s partner (7, 8). Adolescents expressed a need for adequate information on reproduction, conception, and contraception as receiving such information was more acceptable among married members of the community, including the married adolescents (1, 10, 18). Most of the information they knew about contraception and abortion was based on what they heard from parents, partners and their peers, and some information from schools; information mainly comprised of very basic knowledge of the commonest methods (2, 8, 9, 11, 18, 21). Family planning, contraception and abortion information, and services were more acceptable among married people compared to single adolescents (10, 11, 16, 18), but very few of the adolescents are married.

#### Sources of information

Parents, health workers and teachers emerged *as trusted sources* of information. While parents were viewed as trusted and preferred sources, teachers were trusted but not preferred by the adolescents (2, 8, 9, 10, 11, 15, 16, 18, 19). *Parents* were also influencers for contraception use and for seeking abortion (1, 3, 4, 6, 7, 8, 10, 13, 14, 16, 17, 21). The adolescents wished their parents gave them more information as they believed it was authentic (7, 8, 9, 11, 16, 17, 18, 19). The mothers, in particular, were singled out as influencers of abortion. Some parents acknowledged that teaching sexual and reproductive health to adolescents bordered on incest (7) and they were in favour of traditional teachings during initiation ceremonies or before marriage (19). Often, parents only had a once off teaching called “the talk” and would never revisit the discussion (7). From other stakeholders such as the community members, churches and schools, the parents were seen as primary educators who should lay the foundation on the topic of sexual and reproductive health. Then the others, such as teachers in schools merely build upon that (8, 9). *Teachers*, although viewed as a highly trusted source of information were not preferred because the information from them was seen as inadequate and not comprehensive enough, particularly when it came to contraception and abortion (6, 7, 8, 18).

Apart from parents, other family members including aunties, uncles, brothers, sisters, and cousins were available, but not a preferred source of information. However, they were influencers of abortion decisions (7, 8, 11, 16, 17, 18, 19,). *Grandparents* were seen as confidants, but this was seen as slowly reducing (2, 19) because the peers seemed more acceptable than grandparents. The *partners* of the adolescent, usually male were also viewed as a significant influencers for seeking contraception and abortion. They were also considered as confidants, as well as financial supporters, especially if they decided to terminate the pregnancy. The male partners, usually older also gave the young girls information to influence their decision (1, 2, 3, 6, 7, 8, 10, 13, 16, 17, 18) indicating that gender and power relations played a role in decision making around contraception. The peers were major influencers of contraception and seeking an abortion (4, 5, 7, 9, 10, 11, 15, 19, 21). They were a preferred and most common source of information. Peers were also relied upon as confidants (2, 7, 8, 9, 11, 14, 16, 18, 19).

For the boys, *television/ media/ pornography* was also cited as sources of information although these were not preferred sources. However, they were more easily accessible, especially for boys, because they did not need to have a conversation and it was less embarrassing compared to speaking to parents, teachers or health workers. Adolescent boys noted that porn was not morally right and that it led to an increase in sexual activity, violence, and unplanned pregnancies. Despite these views, they opted for the internet (pornography) as a source of information while the females went to the health facility or consulted from their peers and female relatives (7, 8, 11, 18, 19). The boys received more information about condoms compared to girls who received more information about family planning and pregnancy information (8). Generally, males had less knowledge compared to females about contraception and abortion, and they knew condoms or merely the most common methods used in their community (11).

Other sources of information that emerged were *Youth Unions* that went around visiting communities and schools (18). *Churches* were a source but mostly during couples counselling before a wedding and were viewed as a good source of information (18). *Community leaders* were also a source of information that was not preferred by adolescents (18).

*Traditional healers/ ceremonies* were seen to be more affordable than the clinics especially when it came to sexually transmitted infection treatment and abortions. Visitations to traditional healers typically took place during initiation ceremonies or just before marriage (9, 19). Both male and female adolescents agreed that traditional teachings were essential and they appreciated them more if the teacher was of the same gender (9, 19). However, this exchange would be one-sided, more like they were giving instructions without dialogues (19). The customs prevented the sharing of such information in the homes, but it was more acceptable from the traditional healers (9, 10).

### Attitudes towards contraception and abortion

#### Reactions to pregnancy and reasons for abortion

Most of the adolescents, in and out of school expressed the need to be pregnant after marriage to avoid the social stigma of being a mother without marriage, the abortion brought about when more shame than that which was attached to the pregnancy (3, 14, 17). Therefore it was better to fall pregnant than to opt for an abortion. However, with increasing age, it was seen as more difficult to speak to family members about pregnancy because the family members saw their young girls as more capable of making better decisions (2). Some even experienced violence from their families, thereby propagating abortion as a ‘quick fix’ if one fell pregnant. Fear of violence also propagated confiding in peers when the adolescents fell pregnant, increasing the chances of making decisions based on misconceptions or incomplete information (14). Unfortunately, female adolescents had the responsibility of falling pregnant put on them more (5), and they faced more challenges associated with early pregnancy compared to the boys.

#### Abortion

A relationship between an abortion decision and educational level was noted in the review. Adolescents who opted for abortion felt their future would be protected by securing an education and more economic and social empowerment because of not raising a child. They also thought they would preserve their social standing if they were not pregnant (3, 10, 14, 15, 20). Adolescents with higher education levels experienced fewer pregnancies (14). They also knew more about contraception compared to those with lower education levels (15). Emergency contraception was not covered in school sexuality education (7). Adolescents with more education had more abortions compared to those with less education (14). They resorted to unsafe abortion due to lack of information about safe abortion (17). Adolescents who were out of school mainly received information from family, media and the community and not being in school made them more susceptible to manipulation by some men in their lives, as they met their financial and socio-economic needs and demanded sex from them (19).

Some of the female teens opted for abortion because they were raped or because they had sexual intercourse because they felt like they had no other choice (4, 6). In general, induced abortion was more acceptable among married men and women; men were more likely to be the decision makers (13, 10, 21). Among the adolescents, the more sexually active ones knew more about abortion and the different ways it can be achieved. Also, the ones who were more educated even knew more about abortion methods, and sources of the procedure too (15). The cost was usually dependent on how far the pregnancy had developed, meaning that it was more expensive the more developed the pregnancy was (17). Morally, adolescents viewed abortion as a sin and that it was wrong (3, 6, 7).

Adolescents mentioned that they used or would use contraception to avoid pregnancy. However, some indicated that they would not use it because they needed to prove to their partners and their communities that they were fertile. Proving fertility was more common among adolescents who had recently gotten married (1, 5, 13, 15). It should be noted that lack of access is a significant reason why adolescents do not use contraception. However, fear of infertility hinders them from opting for these services, despite their availability. Other reasons included side effects (6), because they were told not to by their partners or they tried to impress their partners or because they wanted to fall pregnant so that he would marry them (4, 14, 16).

### Practices (contraception and abortion)

#### Usage/ contraception methods

The common methods used by adolescents were condoms, mostly among the boys (1, 4, 5, 11, 15, 16, 17, 18, 20, 21). It was more acceptable for the boys to purchase the condoms compared to the girls. Pills (18, 20, 21), sterilizations- in certain parts of the world, usually India (11, 13, 18, 20) and injections (18, 21) were also used. Emergency contraception was used but was not encouraged unless one was raped (7). Adolescent girls mentioned that the best way not to fall pregnant was through abstinence (14). Modern methods were also more common among married adolescents (10, 12, 13). Traditional methods were also fairly common and used but more common in rural areas (1, 13, 15, 17, 18, 21).

#### Sources of abortion or practitioners

Some of the sources or practitioners of abortions are listed in Table 2. For the girls in rural areas, but not limited to these, traditional healers were mostly consulted, while teens from the urban areas mostly went to the health facility (usually illegal procedure), to a pharmacy or tried to induce the abortion themselves based on advice from their mothers, relatives or peers (Table 2).

**Table 2:** Sources, practitioners of abortion

Sources, Practitioners of abortion	Reference
Older women, mothers, aunties	(10,17)
Pharmacists,	(20)
Untrained Paramedical workers	(10)
Traditional healers	(2,9,10,17,21)
Trained practitioner (trained to conduct an abortion	(4)
Doctors, usually private practice	(10,13)
Obstetricians	(10)
Other health providers	(2,4,17,20,21)
Traditional Birth Attendants	(2,13)
Self	(2,17,20)

#### Abortion methods

Numerous abortion methods were captured in the review of the literature. These methods cut across environment or education status or even age of the adolescent. Most of them tried extreme methods based on hearsay advice from their information sources such as mothers, relatives, friends and some health workers. Most of the girls tried these methods because they merely ‘heard’ that they worked (Table 3).

**Table 3:** Abortion Methods

Method	Reference
Pain medication, Sedatives, Anesthesia, Antibiotics	(2,10,17,21)
Chlorine, White Quinine,	(2,15,17,20,21)
Roots (cassava- cyanide), *Aloe Vera*, Castor oil, Ashes	(2,10,14)
Ground tobacco, salt water & sugar solutions, parsley oil,	(15)
Laxative, brandy, and other drinks	(15)
Andrew liver salt, hot pepper salt	(14)
Physical removal (with cassava root), chilli, or pawpaw	(2,14,17)
Curetting / D&C, physical charms	(4,13,20,15)
Boiled beer, tea, Fanta, coca cola	(2,14)
Washing powder/soap	(2,15,21)
Crushed bottles, battery acid, methylated spirit	(2)

Female adolescents often needed permission to use contraception, and when they agreed, they experienced less tension (14, 18). The females also rarely bought condoms over the counter compared to boys who usually did, irrespective of age or education level (5, 20). Adolescents from rural areas face a transportation barrier as they had to travel long distances to access health services and the services were not that good because, at the facilities, they usually faced shortages of health workers (6, 8, 9, 10). So the traditional healers were more accessible to them, and their advice was even more acceptable due to the strong influence of traditional customs (9, 10). Mainly, the husbands also influenced the adolescent women’s’ contraception decisions (10). Among married adolescents, contraception was used for spacing (4, 16, 18). A few girls cited limited access services due to incarceration, while some did not use contraception and family planning because of ignorance, myths, forgetting or due to alcohol intake (4, 6).

## Discussion

We set out to conduct a systematic review on knowledge, attitudes and practices around contraception and abortion among adolescents. The review was undertaken to identify any associated related gaps that may generally be critically important to increase the understanding of the sexual and reproductive health dynamics that many adolescents in LMICs face.

### Knowledge

Adolescents had limited and usually incomplete information about contraception and abortion. We learnt that incorrect information and misconceptions were usually from unreliable sources rather than from trusted sources of information. The lack of unreliable sources was consistent with the findings of other studies [13–15]; that discovered that most of the data received by the young women were often reinforced by the misconceptions and wrong information. Another study pointed out that parents did not give such information because they did not receive it; thus they did not know how to package it [9]. This could be one fundamental reason why the parents did not provide their adolescents with sufficient information, even though the adolescents wanted to receive trusted information from them.

It was interesting to note that the adolescents had various trusted sources of information; health workers, parents, and teachers. While their partners [13] were seen as people they could share information with; adolescents found themselves receiving information more from their peers and television, printed material and the internet (pornography). There was a strong indication that adolescents received less of the accurate, trusted information and more of the less reliable information because it was more readily available. This finding is consistent with other studies. Also, evidence has shown that low knowledge has direct implications on behavior, as it is fueled by wrong or incomplete information [15, 16] and that strong parental relationships coupled with information sharing about risky behavior have shown a decrease in adolescent pregnancy risk [17]. The importance of involving parents in adolescent sexual and reproductive health issues should not be underrated, as this is a critical strategy for improving adolescent sexual and reproductive health. Such strategies must also be mindful of the cultural discomfort that arises when parents talk about contraception and abortion with their children.

### Attitudes

In our reviewe, we discovered that abortion was typically more acceptable among the married adolescents compared to the single ones [18] and the decision makers were most often the male partners. Similarly, health facility abortions but with untrained abortion practitioners and traditional healers were reported as the most common sources with easier accessibility [13]. Adolescents reported many extreme methods of abortion that they used or had heard about from their mothers, other family members or peers with very few saying the actual correct procedure that is done in the health facility. There is a strong suggestion here of the absence of right and accurate information for the adolescents evident by the extreme measure that the young women underwent to terminate a pregnancy. The right information of the on safe procedures and the circumstances under which they can be accessed was not shared with them [19].

In the review, it was noted that even though adolescents had little information, the boys were less knowledgeable about contraception and abortion than the girls. However, it was also pointed out that adolescent girls faced more stigma when pregnant or if they terminated a pregnancy. The fear of shame due to abortion was far much higher than the stigma due to pregnancy, potentially a reason why adolescents did not opt for safe abortion when they fell pregnant. With strong social constructions about the immorality of abortion [20], adolescents felt it was more acceptable to be pregnant. Other studies have cited abortion stigma as a challenge for many women, because of the negative label that they are given by the community around them, and by themselves too. Stigma was noted as a significant reason for clandestine abortions; to maintain secrecy.

The attitude of the health workers also affected whether the adolescent would feel free enough to access information from them or not, though they were seen as trusted sources, many adolescents reported that they did not feel like they received adequate information. As a response to the health care provider crisis in Australia, training general practitioners to be more attentive to youths’ to youth has been under strong consideration [21]. The extent to which health care providers were helpful and unbiased in their service provision was different among the different adolescents. While some experienced positive attitudes, others were treated in a cold, arrogant and judgmental manner. Negative attitudes were typical among many health care providers, especially in conservative settings. A review of studies in LMICs showed that they were willing to provide only condoms to the adolescents because the other methods were not appropriate for them [22].

### Practices

Adolescents contraceptive use was typically low, but they practiced the most common methods of contraception known because these were the ones they had easier access to as was found in other studies too [9, 13, 22]. While this review found that traditional methods were preferred, other studies have indicated that the traditional methods were used because they were more accessible than modern contraceptives [13, 19]. The low usage is mirrored from the lack of information about contraception and abortion among adolescents. It was cited in the review that girls who had older partners were not decision makers for contraception use and abortion. They also received information only as it related to the decision of their partner. The lack of autonomy made the girls more vulnerable to suggestion, whether this was good for them or not. Emphasis is made on the gendered differences in the consequences of low knowledge as the girls suffer worse health outcomes compared to the boys. This too has an impact on how interventions targeted towards adolescents should be designed.

The interplay between gender, environment, education status and marital status with adolescent access to information and services was seen too in other studies [9, 18, 23]. These findings indicate that adolescents are different and that they have different needs depending on their local context. Many studies look at adolescents as one homogenous group of young people with similar needs, and this leaves out many in the targeted interventions. Other studies have indicated that more education increased the likelihood of contraceptive usage [24]. This could be because most targeted interventions are done with school going adolescents [25]. We noted that rural adolescents faced more barriers to accessing information and services compared to the urban. The urban adolescents had more access to methods available at pharmacies, studies in China indicated, and that urban youths had higher rates of induced abortions compared to the rural adolescents [18]. Both these findings could suggest underreporting of the traditional methods used among the adolescents. It was also much less surprising to find that the married adolescent had more access to sexual and reproductive health information and services compared to the single ones [9, 22]. The review also showed that the more educated adolescents were more knowledgeable about services and issues of conception compared to those who had not been to school entirely and those who had been to school but did not complete their primary education.

This study was not limited to purely qualitative studies but included mixed methods designs too to obtain richer findings. The analytical approaches used in these studies were scrutinized before being included in the analysis. In addition to this, the review also followed a clear analysis that yielded accurate findings. There may have been biased in the definition of knowledge, perceptions, attitudes and practices by adolescents, and more inner individual experiences may have been missed due to the nature of the qualitative inquiry, however, the information outlined is still beneficial to inform current policy on adolescents. Another limitation of this study was the combination of wording used in the keyword search as this might have left out some vital experiences in other literature. However, those findings may have been similar to the ones included in the study as data was similar across countries and regions.

## Conclusion

In conclusion, adolescents face a lack of access to useful information about contraception and abortion as observed in the studies reviewed in this study. Lack of access was propagated mainly by the information sharing norms that exist around sexual and reproductive health. Although interventions have been made in the past, targeting adolescents, lack of information remains a challenge; suggesting severe limitations in prior interventional approaches. Without knowledge, many of them still resort to dangerous solutions when most of these services are accessible, another signal of both system and structural failures inherent in existing service systems. This calls for not only a re-think in the existing strategies but also on who are the other key stakeholders who must take much more critical roles, also keeping in mind the significant differences in barriers among the different categories of adolescents. The reviewed studies suggest past severe limitations in the access to safe and effective methods of contraception which is a vital component of the reproductive health status of young people in developing countries. This further suggests a need for an urgent response in addressing the “unmet need” for contraception, abortion information, and services as a moral and public good for adolescents. In doing so, we argue that holistic approaches and interventions which target broader stakeholder involvement are also critically needed but must be evidence-based taking into account differential contexts and contrasts while remaining with one wholesome, consistent and appropriate message that promote the total well-being of the adolescents.

## Abbreviations

LMICs: ᅟ
