# Correction to: Contraception and abortion knowledge, attitudes and practices among adolescents from low and middle-income countries: a systematic review

**DOI:** 10.1186/s12913-019-4297-5

**Published:** 2019-07-02

**Authors:** Margarate Nzala Munakampe, Joseph Mumba Zulu, Charles Michelo

**Affiliations:** 1 Department of Health Policy and Management, School of Public Health, University of Zambia, Nationalist Road, P.O Box 50110, Lusaka, Zambia; 2 Department of Health Promotion and Education, School of Public Health, University of Zambia, Lusaka, Zambia; 3 Department of Epidemiology and Biostatistics, School of Public Health, University of Zambia, Lusaka, Zambia; 4 Strategic Centre for Health Systems Metrics and Evaluations (SCHEME), Department of Epidemiology and Biostatistics, School of Public Health, University of Zambia, Lusaka, Zambia

**Correction to: BMC Health Serv Res**

**https://doi.org/10.1186/s12913-018-3722-5**

In the original publication of this article [1], there are several errors. The original sentences and corrections are listed below:

1. Some parents acknowledged that teaching sexual and reproductive health to adolescents bordered on incest (7) and they were in favour of traditional teachings during initiation ceremonies or before marriage (19)

Change “bordered on incest” to “were uncomfortable”, and delete citation 7.

2. Apart from parents, other family members including aunties, uncles, brothers, sisters, and cousins were available, but not a preferred source of information. However, they were influencers of abortion decisions (7, 8, 11, 16, 17, 18, 19,).

Delete citation 7

3. Emergency contraception was not covered in school sexuality education (7)

Change “was not covered” to “scarcely covered”.

4. Morally, adolescents viewed abortion as a sin and that it was wrong (3, 6, 7)

Change “adolescents” to “some adolescents”, and keep citation 3,6,7.

5. In our reviewe, we discovered that abortion was typically more acceptable among the married adolescents compared to the single ones [18] and the decision makerswere most often the male partners

Change “in our reviewe” to “in our review”.
